# Impact of lumacaftor/ivacaftor on the bacterial and fungal respiratory pathogens in cystic fibrosis: a prospective multicenter cohort study in Sweden

**DOI:** 10.1177/17534666241254090

**Published:** 2024-05-23

**Authors:** Mahasin Al Shakirchi, Kimmo Sorjonen, Lena Hjelte, Lena Klingspor, Peter Bergman, Petrea Ericson, Marcus Svedberg, Ulrika Lindberg, Christine Hansen, Isabelle de Monestrol

**Affiliations:** Stockholm CF Centre, Karolinska University Hospital Huddinge, Stockholm, Department of Clinical Science, Intervention and Technology, Sweden; Division of Pediatrics, Karolinska Institutet, Alfred Nobels Allé 8, Stockholm 171 77, Sweden; Division of Psychology, Department of Clinical Neuroscience, Karolinska Institutet, Stockholm, Sweden; Stockholm CF Centre, Karolinska University Hospital Huddinge, Stockholm, Sweden; Department of Clinical Science, Intervention and Technology, Division of Pediatrics, Karolinska Institutet, Stockholm, Sweden; Division of Clinical Microbiology, Department of Laboratory Medicine, Karolinska University Hospital Huddinge, Karolinska Institutet, Stockholm, Sweden; Department of Infectious Diseases, The Immunodeficiency Unit, Karolinska University Hospital Huddinge, Karolinska Institutet, Stockholm, Sweden; Division of Clinical Microbiology, Department of Laboratory Medicine, Karolinska University Hospital Huddinge, Karolinska Institutet, Stockholm, Sweden; Department of Respiratory Medicine, Sahlgrenska University Hospital, Gothenburg, Sweden; Department of Pediatrics, Institute of Clinical Science, Gothenburg University, Sweden; Lund CF Centre, Skåne University Hospital, Lund, Sweden; Lund CF Centre, Skåne University Hospital, Lund, Sweden; Stockholm CF Centre, Karolinska University Hospital Huddinge, Stockholm, Sweden; Department of Clinical Science, Intervention and Technology, Division of Pediatrics, Karolinska Institutet, Stockholm, Sweden

**Keywords:** bacteria, cystic fibrosis, fungi, lumacaftor/ivacaftor

## Abstract

**Background:**

A significant decline in pulmonary exacerbation rates has been reported in CF patients homozygous for F508del treated with lumacaftor/ivacaftor. However, it is still unclear whether this reduction reflects a diminished microbiological burden.

**Objectives:**

The aim of this study was to determine the impact of lumacaftor/ivacaftor on the bacterial and fungal burden.

**Design:**

The study is a prospective multicenter cohort study including 132 CF patients homozygous for F508del treated with lumacaftor/ivacaftor.

**Methods:**

Clinical parameters as well as bacterial and fungal outcomes 1 year after initiation of lumacaftor/ivacaftor were compared to data from 2 years prior to initiation of the treatment. Changes in the slope of the outcomes before and after the onset of treatment were assessed.

**Results:**

Lung function measured as ppFEV1 (*p* < 0.001), body mass index (BMI) in adults (*p* < 0.001), and BMI *z*-score in children (*p* = 0.007) were improved after initiation of lumacaftor/ivacaftor. In addition, the slope of the prevalence of *Streptococcus pneumoniae* (*p* = 0.007) and *Stenotrophomonas maltophilia* (*p* < 0.001) shifted from positive to negative, that is, became less prevalent, 1 year after treatment, while the slope for *Candida albicans* (*p* = 0.009), *Penicillium* spp (*p* = 0.026), and *Scedosporium apiospermum* (*p* < 0.001) shifted from negative to positive.

**Conclusion:**

The current study showed a significant improvement in clinical parameters and a reduction of some of CF respiratory microorganisms 1 year after starting with lumacaftor/ivacaftor. However, no significant changes were observed for *Pseudomonas aeruginosa, Staphylococcus aureus*, or *Aspergillus fumigatus*, key pathogens in the CF context.

## Introduction

Cystic fibrosis (CF) is one of the most common life-limiting hereditary diseases in Caucasian populations. The main defect in CF is a dysfunctional CF transmembrane conductance regulator (CFTR) protein coded by the *CFTR* gene localized on the long arm of the seventh chromosome.^1^ Despite the early discovery of the gene in 1989 and progress in understanding the pathophysiology of the disease, it was not until 2012 that the first substance to correct the basic defect in CF, ivacaftor, was approved.^2–4^ Since then, several mutation-specific CFTR modulators have been developed with a wide variation in efficacy. Lumacaftor/ivacaftor consists of one potentiator (ivacaftor) and one corrector (lumacaftor) and is approved for people with CF (pwCF) homozygous for F508del.^5^ Treatment with lumacaftor/ivacaftor has shown a significant though modest improvement in lung function measured as the percentage of predicted forced expiratory volume in 1 second (ppFEV_1_) ranging between 2.6% and 4.0% and a significant reduction in pulmonary exacerbation rate with 30–39% compared to placebo.^6^ Similarly, a real-life multicenter study from France showed an improvement in lung function to the same extent, an increase in body mass index (BMI) and a decrease in the number of courses of intravenous antibiotic treatments.^7^

The intriguing question is whether the reduction in pulmonary exacerbations and antibiotic courses reflects an antimicrobial activity. Up to date, there are limited data published on the impact of lumacaftor/ivacaftor on microbiological outcomes. Singh *et al*. reported that the time to first acquisition of *Pseudomonas aeruginosa* or *Staphylococcus aureus* was significantly delayed in pwCF treated with lumacaftor/ivacaftor compared to the nontreated group.^8^ Quantitative polymerase chain reaction (qPCR), a nonconventional microbiological detection method, was used to assess the microbiological response to lumacaftor/ivacaftor therapy.^9–11^ Neerincx *et al*. demonstrated no significant changes in the bacterial composition nor the relative abundance of *P. aeruginosa* after lumacaftor/ivacaftor initiation.^9^ Likewise, Enuad *et al*. reported no changes in the diversity or pathogen abundance expect in pwCF not chronically colonized with *P. aeruginosa*,^10^ while Graeber *et al*. reported decreased bacterial load and increased diversity but no changes in the pathogen abundance.^11^ *In vitro*, an antibacterial property of lumacaftor/ivacaftor has been reported.^12^ A combination of polymyxin B and lumacaftor/ivacaftor has been shown to have a synergistic effect against polymyxin B resistant *P. aeruginosa* not possible to achieve by each drug alone.^12^ Ivacaftor is the most studied CFTR modulator. Studies on the impact of ivacaftor in pwCF with the gating mutation G551D on the bacterial outcomes demonstrated conflicting results. For example, it has been reported that the odds of *P. aeruginosa* detection were significantly lower after introducing ivacaftor.^13^ Furthermore, the density of *P. aeruginosa* using qPCR and 16S rRNA gene sequencing as well as inflammatory parameters in sputum such as neutrophil elastase, interleukin (IL)-8, and IL-1b rapidly declined after ivacaftor initiation.^14^ In addition, a potent antibacterial activity of ivacaftor against *S. aureus* has been reported.^15^ Several studies investigated the impact of elexacaftor/tezacaftor/ivacaftor on the microbiological outcomes showed a reduction in the positive sputum culture and bacterial load, an increased microbiome diversity and reduction in the isolation rate of *P. aeruginosa* and *S. aureus* as well as cytokines after introducing elexacaftor/tezacaftor/ivacaftor.^16–22^ In contrast, other researchers have not been able to detect alterations in the bacterial load nor reduced levels of inflammatory parameters after introducing ivacaftor.^23,24^ The reasons for these discrepancies remain elusive but could be related to different study design or treatment length.

Studies regarding the impact of CFTR modulators on fungi are even more scarce. Given the drug-drug interaction between CFTR modulators and azoles, the most common antifungal class agents used in CF, it is of a great interest to examine the potential antifungal activity in the era of CFTR modulators, a topic emphasized by CF researchers.^25^ From a theoretical perspective, restoring CFTR function may improve the ability to clear *Aspergillus fumigatus* conidia and prevent the exaggerated and aberrant inflammatory response demonstrated in CF.^26^ In fact, an *in vitro* experiment has shown that peripheral blood mononuclear cells and polymorphonuclear cells isolated from pwCF produced significantly lower levels of the reactive oxygen species as a response to *A. fumigatus* exposure when treated with CFTR modulators (ivacaftor, lumacaftor, ivacaftor/lumacaftor) compared to untreated cells.^27^ Interestingly, the authors found that reactive oxygen species was reduced also in phagocytes treated with CFTR modulator from healthy controls, suggesting an antifungal effect.^27^ On the other hand, a recent study on mycobiome reported no changes in the fungal diversity or abundance in pwCF treated with lumacaftor/ivacaftor.^10^ While studies on ivacaftor showed that the prevalence of *A. fumigatus* was significantly reduced after ivacaftor treatment.^13,28,29^ Likewise, an observational retrospective study in pwCF treated with elexacaftor/tezacaftor/ivacaftor showed a substantial reduction in *A. fumigatus* isolation in sputum in preliminary result.^30^ Nonetheless, colonization with *A. fumigatus* was more prevalent in pwCF treated with lumacaftor/ivacaftor compared to the nontreated group, however; this was inconsistent in the second year of the follow-up period.^31^

As the predominant symptoms in CF derive from the microbial burden and the subsequently triggered inflammation, it is of great interest to investigate the impact of CFTR modulators on the bacterial and fungal microorganisms in the CF respiratory tract. Here, we aimed to study the effects of lumacaftor/ivacaftor on the detection of key CF pathogens.

## Methods

### Study population

The current study is a prospective multicenter cohort study. Lumacaftor/ivacaftor was introduced in Sweden in 2018. Due to the high cost of lumacaftor/ivacaftor, authorities demanded a follow-up of the treatment efficacy using the National Swedish CF registry. PwCF attending Stockholm, Lund and Gothenburg CF centers participated in the study covering 93% of the eligible patients. Patients from the fourth CF center, Uppsala CF center (13 patients), were not included due to lack of microbiological data as most of them had shared care. The inclusion criteria were pwCF: (1) Older than 5 years, (2) Homozygous for F508del, (3) Accomplishing complete microbiological data during the study period, (4) Maintaining the treatment for at least 1 year, and (5) Unexposed to lumacaftor/ivacaftor before the start of enrollment. The study period was 3 years comprising 1 year after the initiation of lumacaftor/ivacaftor and the preceding 2 years (2016–2021). Each patient was used as its own control before and after the initiation of treatment.

**The National Swedish CF registry:** The National Swedish CF registry is a Swedish health quality registry containing data on demographic, clinical parameters, treatment, and microbiological outcomes with 100% coverage of the CF population treated with CFTR modulators in Sweden. According to a nationally developed program, the first year after lumacaftor/ivacaftor initiation, pwCF were followed up by five mandatory scheduled visits (1, 3, 6, 9, and 12 months after lumacaftor/ivacaftor initiation). At each visit, respiratory cultures were planned to be obtained.

### Study variables

For each year of the study period demographic, clinical characteristics, microbiological outcomes, and treatment burden were retrieved from the National Swedish CF registry. The clinical characteristics were ppFEV1 and percent predicted forced vital capacity (ppFVC), BMI in adults (⩾18 years of age) and BMI *z*-score in children (<18 years). The microbiological outcomes included the number of bacterial and fungal cultures obtained each year, and the bacterial and fungal growth as well as *S. aureus* and *P. aeruginosa* colonization’s status according to Leeds criteria.^32^ Treatment burden (defined as the number of intravenous antibiotic courses, continuous oral antibiotic treatment, nebulized antibiotic use, azithromycin use) and inflammatory parameters [C-reactive protein (CRP), erythrocyte sedimentation rate (ESR) and immunoglobulin G levels (IgG)] were evaluated. Sweat chloride and Lung Clearance Index (LCI), performed only in pwCF whose ppFEV1 was ⩾80%, were performed before the initiation of lumacaftor/ivacaftor and 3 and 12 months after. For pwCF attending Stockholm CF center all included patients or their legal guardians signed informed consents for an extended evaluation. For this subset, additional microbiological data including the number of positive cultures for each bacterium and fungus for each year of the study period, *A. fumigatus* specific immunoglobulin E (IgE) and immunoglobilin G (IgG) as well as specific antibodies for *P. aeruginosa* (alkaline protease, elastase, exotoxin A) and *S. aureus* (alfatoxin and teichonic acid) were available from the medical records and included in the analyses.

Eligible patients started with lumacaftor/ivacaftor shortly after or at the same time as the annual assessment. The studied bacteria were *P. aeruginosa, S. aureus, Achromobacter species, Stenotrophomonas maltophilia, Haemophilus influenzae, Serratia Marcescens, Moraxella catarrhalis*, and *Streptococcus pneumoniae.* The studied fungi were *A. fumigatus, Scedosporium apiospermum, Candida albicans, C. dubliniensis, C. glabrata, C. parapsilosis, Exophiala dermatitidis*, and *Penicillium spp.*

### Microbiological methods

Sputum was treated with sputolysin (dithiothreitol + buffer) to solubilize the sputum-sample. Next, 10 μl were plated onto different agar-plates (hematin agar, gentian violet blood agar, cysteine lactose electrolyte deficient agar, pseudomonas-agar and cepacia-agar) in serial dilutions (1×10E3 – 1X10E8 CFU/ml) and incubated in ambient air or carbon dioxide (37°C), depending on the required growth conditions. The plates were evaluated for bacterial growth on day 1 and day 2. Bacterial identification was performed using a Microflex LT MALDI-TOF system in the linear positive ion-mode using FlexControl 3.3 software (Bruker Daltonics) as outlined in PMID: 37611433. The Bruker bacterial test standard was used as a calibrator for each sample run. Each spectrum was compared with a reference spectrum from the database provided from the manufacturer, using the software MALDI Biotyper 3.0 (Bruker Daltonics). Subculturing and testing for antibacterial susceptibility were performed according to methods outlined by EUCAST (https://www.eucast.org/).

Yeast and filamentous fungal culture were performed on 2% dextrose blood agar (with chloramphenicol and vancomycin), on Sabouraud’s agar (with chloramphenicol and gentamicin) and on chromogenic medium (CHROMagar™ Candida Plus, CHROMagar Microbiology, Paris, France). Dextrose blood agar and chromogenic agar were incubated at 37°C for 7 days, while Sabouraud agar was incubated at 30°C for 7 days. For the identification of Candida species, color and appearance of yeast colonies on CHROMagar CandidaTM Plus was evaluated. Green yeast colonies were further identified to species level by BICHRO-DUBLI FUMOUZE^®^ (Fumouze Diagnostics, Levallois-Perret, France) latex agglutination tests or by MALDI-TOF MS. Latex agglutination tests were also used to detect C. krusei.

Filamentous fungal cultures were additionally incubated at 42°C on Potato Dextrose Agar (with penicillin and streptomycin) for 7 days and identified according to their macroscopic and microscopic characteristics.

### Primary and secondary outcomes

The association between time and the outcome variables, that is, the slope, was compared before and after the initiation of treatment. The slope for 2 years preceding the onset of treatment served as a control, indicating the expected development in the outcome variable without treatment with lumacaftor/ivacaftor. The primary outcomes were changes in the slope of the prevalence of CF key microorganisms. CF patients attending Gothenburg CF center were not included in the statistical analysis for yeast due to a change in reporting policy. The secondary outcomes were the impact of lumacaftor/ivacaftor on the slope of other clinical parameters including lung function, BMI in adults, BMI z-score in children, LCI, sweat chloride, and treatment burden.

Furthermore, a sensitivity analysis was performed to address the robustness of our analysis. PwCF who provided less than three respiratory cultures annually, for bacteria and fungi respectively, for each year of the study period were excluded. For Stockholm’s CF patients, an additional analysis was performed where the annual number of positive cultures for each pathogen was included.

The reporting of this study conforms to the Strengthening the Reporting of Observational Studies in Epidemiology (STROBE) statement.^33^

### Statistical methods

Employing R 4.1.0 statistical software^34^ and the lmerTest^35^ and lspline^36^ packages, data was analyzed with multilevel piecewise linear spline regression, with a single knot placed at the start of the lumacaftor/ivacaftor treatment. The outcome variables were either continuous, dichotomous, or count variables. (1) Continuous outcome variables were analyzed with linear models. Here, the association with time, that is, the slope, indicates how much the outcome variable is predicted to change on its scale per year. (2) Dichotomous outcome variables were analyzed with logistic models. Here, the slope indicates change in the logarithm of the predicted odds for outcome per year. This can be transformed to change in the probability, that is, the predicted prevalence, for the outcome per year. (3) Count variables were analyzed with Poisson models. Here, the slope indicates change in the logarithm of the predicted count per year. This can be transformed to change in the predicted count per year.

The linear models included both a random intercept, accounting for individual differences in the outcome variable at the start of treatment, and a random slope with a single knot, accounting for individual differences in the slope both before and after the start of treatment. The logistic and Poisson models included a random intercept. Here, the random slope was omitted due to difficulties for the models to converge.

## Results

### Characteristics of the study population

The number of eligible patients was 162, of which 132 had completed microbiological data and maintained lumacaftor/ivacaftor treatment for at least 1 year. The majority were adults at the start of treatment (60%). Patients contributed with a mean of 6.3 bacterial cultures/year and a mean of three fungal cultures/year. The majority of the subjects were colonized with *P. aeruginosa* at the start of treatment (82%, chronic or intermittent colonization according to Leeds criteria^32^), and 30% were colonized with *A. fumigatus* defined as the presence of *A. fumigatus* in sputum culture ⩾1/year.^37^ The demographic and clinical data were similar at all three CF centers. Characteristics of pwCF at the annual assessment shortly before or at the same time as the start with lumacaftor/ivacaftor are presented in Table 1.

**Table 1.** Demographic and clinical data of pwCF at the annual assessment prior to the start with lumacaftor/ivacaftor.

	Total	Stockholm	Lund	Gothenburg
*N*	132	46	51	35
Mean age, (range), year	23 (6 to 57)	25 (6 to 56)	21 (6 to 57)	24 (6 to 47)
Adults/children<18 years), *n*	78/54	30/16	25/26	23/12
Gender, male/female, *n*	70/62	19/27	30/21	21/14
ppFEV1, mean (range)	81 (21 to 123)	81 (25 to 119)	82 (21 to 123)	78 (31 to 112)
ppFVC, mean (range)	94 (38 to 129)	95 (53 to 127)	92 (38 to 127)	94 (61 to 129)
BMI adults range	21.71 (16.30 to 28.83)	21.98 (16.74 to 28.83)	21.13 (16.30 to 28.36)	21.86 (18.18 to 25.34)
BMI *z*-score in children range	−0.26 (−2.01 to 1.43)	−0.003 (−1.58 to 1.43)	−0.16 (−2.01 to 1.45)	−0.86 (−1.9 to 0.53)
Mean sweat chloride, mmol/L	105	101	107	108
Insulin treated CFRD^a^, *n* (%)	23 (17%)	8 (17%)	10 (20%)	5 (14%)
Treatment burden				
Azithromycin, *n*	43	11	23	9
Inhaled antibiotics, *n*	56	16	24	16
Continuously oral antibiotic treatment	35	18	11	6
Mean number of intravenous antibiotic courses	1.4	1.8	1.2	1.1
Antifungal treatment, *n*	18	6	7	5
Microbiological outcomes				
Number of bacterial cultures	853	297	349	207
Average of bacterial cultures per patient, *n*	6.5	6.5	6.8	5.9
Number of fungal cultures	346	152	125	69
Average of fungal cultures per patient, *n*	2.6	3.3	2.5	2.0
Patients chronically colonized with *P. aeruginosa*,^b^ *n* (%)	53 (40%)	20 (43%)	19 (37%)	14 (40%)
Patients intermittent colonized with *P. aeruginosa*,^b^ *n* (%)	24 (18%)	6 (13%)	16 (31%)	2 (6%)
Patients noncolonized with *P. aeruginosa*,^b^ *n* (%)	55 (42%)	20 (43%)	16 (31%)	19 (54%)
Patients chronically colonized with *S. aureus, n* (%)	52 (39%)	14 (30%)	24(47%)	14 (40%)
Patients colonized with *Achromobacter* spp, *n* (%)	9 (7%)	3 (7%)	5 (10%)	1 (3%)
Patients colonized with *Burkholderia* cepacia Complex^c^, *n* (%)	7 (5%)	2 (4%)	2 (4%)	3 (9%)
Patients colonized with *A. fumigatus, n* (%)	40 (30%)	18 (39%)	14 (27%)	8 (23%)
Patients colonized with *C. albicans, n* (%)	46 (35%)	31 (67%)	15 (29%)	No data

a CF related diabetes.

b According to Leeds colonization criteria for *P. aeruginosa*.

c *B. cenocepacia, B. vietnamiensis*, and *B. multivorans.*

### The impact of lumacaftor/ivacaftor on clinical outcomes

**Figure 1.**
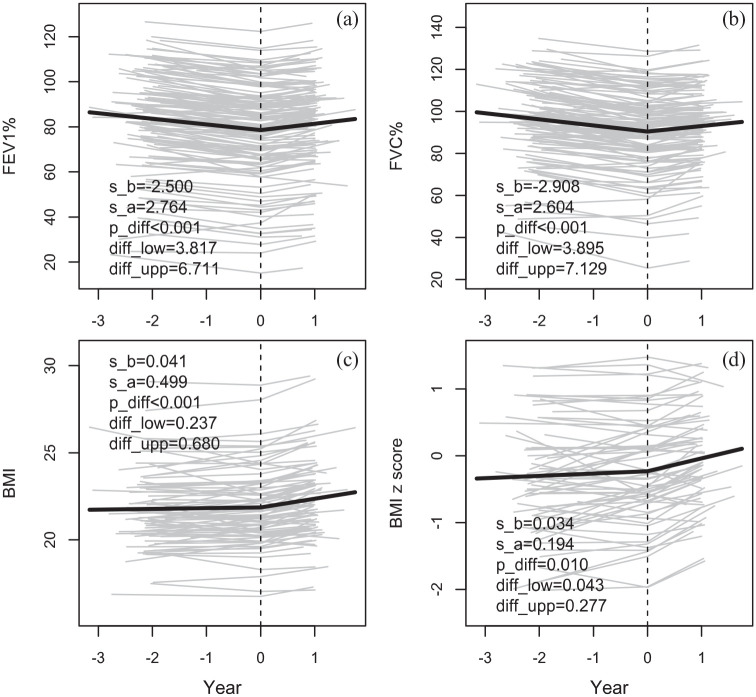
The slope of ppFEV1 (a), ppFVC (b), BMI (c), and BMI *z* score (d) before (s_b) and after (s_a) the start of treatment (Year = 0). Also, the significance (p_diff) and the 95% CI (diff_low- diff_upp) of the change in slope at the start of treatment are given.

The slopes of ppFEV1and ppFVC shifted from negative (−2.5% for ppFEV1 and −2.9% for ppFVC) to positive after start of treatment (+2.8% for ppFEV1 and +2.6% for ppFVC), *p* < 0.001 (Figure 1). Similarly, the slopes of BMI in adults and BMI z-score in children increased significantly from 0.041 to 0.499 (*p* < 0.001) for BMI and from 0.034 to 0.194 (*p* = 0.010) for BMI *z*-score after treatment (Figure 1). The slope of total IgE (*p* = 0.012) and the inflammatory parameters CRP (*p* < 0.001), ESR (*p* < 0.001), and IgG (*p* < 0.001) decreased significantly after the start of treatment. Further, sweat chloride concentrations decreased significantly with a mean of 17 mmol/L, *p* < 0.001. Among individuals with ppFEV1 ⩾ 80 at the start of treatment LCI was predicted to decrease by 0.81 LCI-units per year (*p* < 0.001). Notably, there was no effect of gender or age on the variation of the effect of lumacaftor/ivacaftor on lung function. However, the reduction of sweat chloride concentrations in female participants was more pronounced than in male participants (*p* = 0.016) and no variation in the slopes was observed between age groups.

### The impact of lumacaftor/ivacaftor on treatment burden

**Figure 2.**
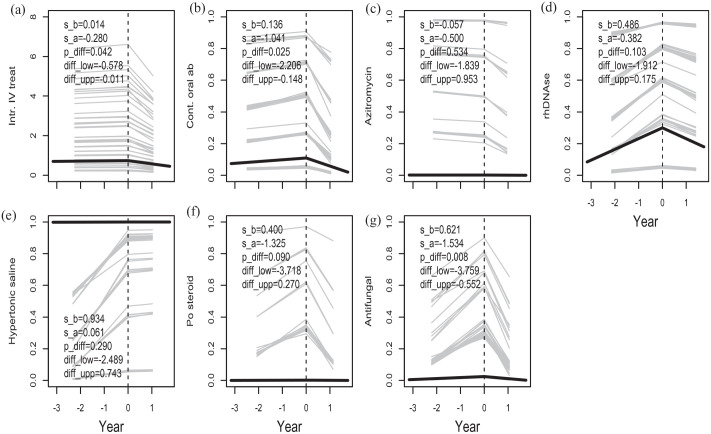
The slope of number of intravenous antibiotic treatments (a), the probability of being continuously treated with oral antibiotic (b), azithromycin (c), rhDNAse (d), hypertonic saline (e), oral corticosteroid (f), antifungal treatment (g). Also, the significance (p_diff) and the 95% CI (diff_low- diff_upp) of the change in slope at the start of treatment are given. The presented slopes before (s_b) and after (s_a) the start of treatment is for the natural logarithm of the count (a) and for the natural logarithm of the odds for outcome (b–g) but predicted values have been transformed to count (a) and probability (b)–(g) for ease of interpretation.

Further, we studied changes in treatment burden before and after lumacaftor/ivacaftor initiation (Figure 2). The number of intravenous antibiotic courses was predicted to increase by 1.4% per year before the start of treatment and to decrease by 24% per year after the start of treatment. This change in slope was significant (*p* = 0.042). Likewise, the odds of being treated continuously with oral antibiotic was predicted to increase by 15% per year before the start of treatment and to decrease by 65% per year after the start of treatment and the difference in slope was significant (*p* = 0.025). The odds of receiving antifungal agents were predicted to increase by 86% per year before the start of treatment and to decrease by 78% per year after the start of treatment. This difference in slope was significant (*p* = 0.008). On the other hand, the concomitant treatment with inhaled antibiotic, hypertonic saline, rhDNAse, azithromycin or oral corticosteroids did not change during the study period.

### The impact of lumacaftor/ivacaftor on respiratory CF microorganisms

**Figure 3.**
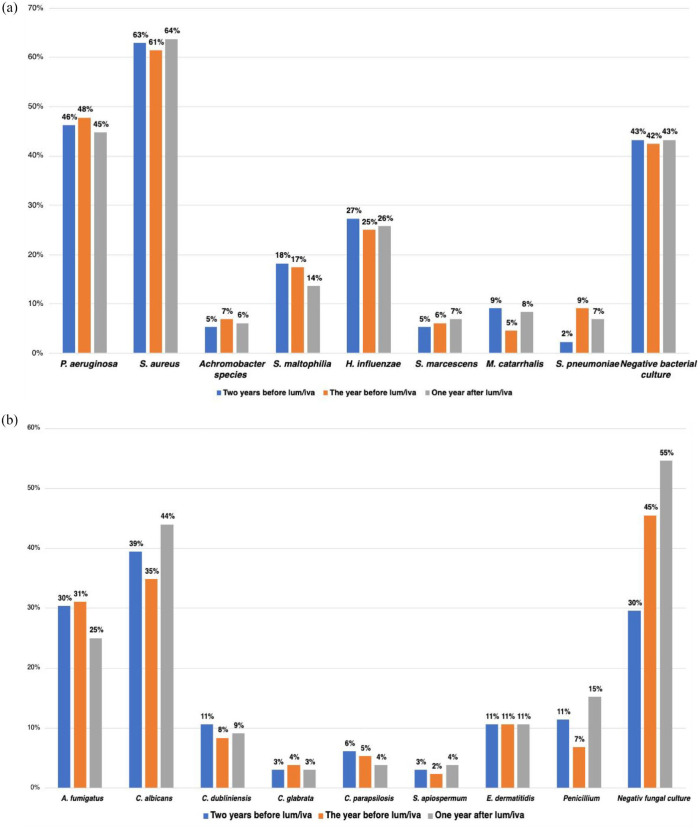
(a) The bacterial prevalence for each year of the study period and (b) The fungal prevalence for each year of the study period.

**Figure 4.**
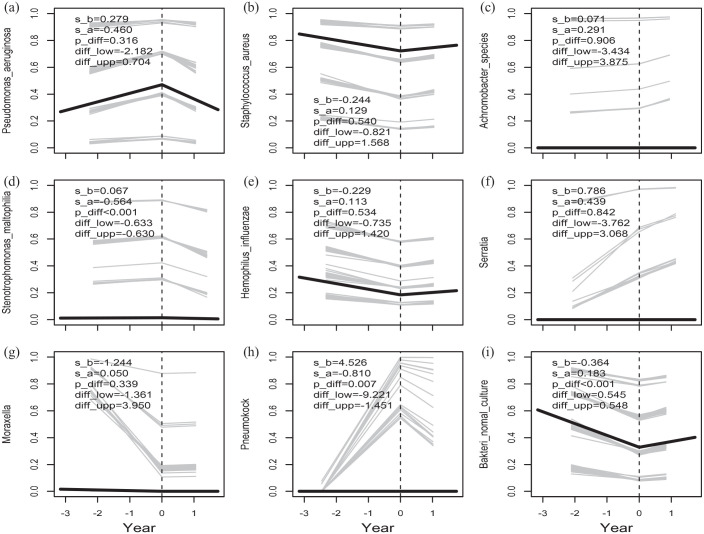
The slope of the probability, that is, the prevalence, of *P. aeruginosa* (a), *S. aureus* (b), *Achromobacter spp*. (c), *S. maltophilia* (d), *H influenzae* (e), *S. marcescens* (f), *M. catarrhalis* (g) and *S. pneumoniae* (h), negative culture for bacteria (i). Also, the significance (p_diff) and the 95% CI (diff_low- diff_upp) of the change in slope at the start of treatment are given.

**Figure 5.**
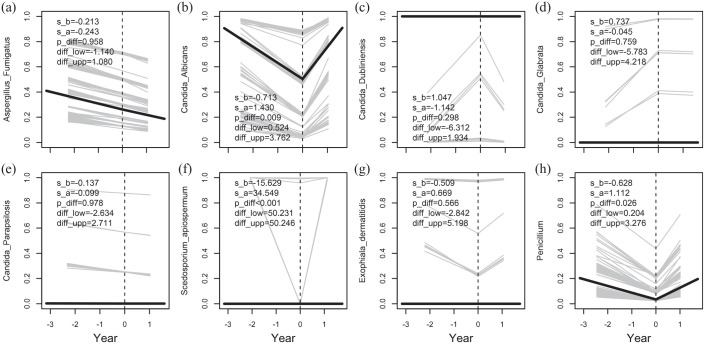
The slope of the probability, that is, the prevalence, of *A. fumigatus* (a), *C. albicans* (b), *C. dubliniensis* (c), *C. glabrata* (d), *C. parapsilosis* (e), *S. apiospermum* (f), *E. dermatitidis* (g) and *Penicillium* spp. (h). Also, the significance (p_diff) and the 95% CI (diff_low- diff_upp) of the change in slope at the start of treatment are given. The presented slopes before (s_b) and after (s_a) the start of treatment are for the natural logarithm of the odds for outcome but predicted values have been transformed to probability for ease of interpretation.

In total 2563 bacterial and 1219 fungal cultures were obtained during the 3-year study period. The prevalence of the bacterial and fungal outcomes for each year of the study period is presented in Figure 3(a) and (b). The most prevalent bacteria were *S. aureus* and *P. aeruginosa*, and the most prevalent fungi were *C. albicans* and *A. fumigatus*. The number of negative bacterial culture remained unchanged, while the number of negative fungal culture increased throughout the study period. The slope of the bacterial and fungal prevalence after lumacaftor/ivacaftor treatment compared to before treatment was assessed, Figures 4(a) and 5. No significant change in the slope of the annual number of obtained bacterial cultures was observed after lumacaftor/ivacaftor initiation. In contrast, the slope of the annual number of obtained fungal culture changed from a predicted decrease by 2.6% per year before the start of the treatment to a predicted increase by 40% per year after the start of the treatment. This change in slope was significant (*p* = 0.004). The slope of the prevalence of *S. maltophilia* (*p* < 0.001) and *S. pneumoniae* (*p* = 0.007) decreased significantly after lumacaftor/ivacaftor initiation. Notably, the slope of prevalence to culture negativity for bacteria, that is, normal culture, increased from a predicted decrease by 31% per year to a predicted increase by 20% per year. No significant changes in the slope of the prevalence of the remaining bacteria were observed. Furthermore, no significant changes in the slope of colonization status and specific antibodies for *P. aeruginosa* and *S. aureus* (available only for pwCF attending Stockholm CF center) were observed during the study period.

For the fungal outcomes, the slope of the prevalence of *C. albicans* (*p* = 0.001), *Penicillium* spp (*p* = 0.026) *and S. apiospermum* (*p* < 0.001) increased after treatment with lumacaftor/ivacaftor. For the remainder of studied fungi, no changes were observed. Regarding the serological analyses, no significant changes were observed in the slope of the levels of *A. fumigatus* specific antibodies (IgE and IgG). Because the very low incidence rate of allergic bronchopulmonary aspergillosis in our cohort, this condition was not studied here.

### Sensitivity analyses

The number of respiratory cultures may impact the results, and to ensure low probability of false negative bacterial or fungal cultures, we performed sensitivity analyses by including only pwCF who had ⩾3 bacterial or fungal cultures annually (*n* = 101). Consistent with the previous analysis, the slope of the prevalence of *S. pneumoniae* was significantly lower with lumacaftor/ivacaftor (*p* = 0.003). Additionally, the slope of the prevalence of *Achromobacter* species was also significantly decreased post lumacaftor/ivacaftor (*p* *=* 0.007). No changes were detected regarding the rest of the bacterial outcomes or on the fungal findings.

### PwCF at the Stockholm CF center

Data on the number of positive cultures for each pathogen of interest for each year of the study period were available for pwCF at Stockholm CF Center (*n* = 46). Further analysis obtained in this subset showed no change of slope of the prevalence of bacterial outcomes. Regarding the fungal outcomes, the slopes of the prevalence of *C. albicans* (*p* = 0.054) and the number of negative fungal cultures (*p* = 0.031) increased significantly after lumacaftor/ivacaftor initiation, while the slope for the prevalence of *C. glabrata* decreased (*p* < 0.001).

## Discussion

This study is a real-life prospective multicenter study on the impact of lumacaftor/ivacaftor on the bacterial and fungal microbiology in CF airways. The cardinal findings were that *S. maltophilia* and *S. pneumoniae* as well as *Achromobacter* species (in the sensitivity test) became less prevalent whereas *C. albicans, Penicillium* spp, and *S. apiospermum* were more frequent after lumacaftor/ivacaftor initiation. Unfortunately, treatment with lumacaftor/ivacaftor did not impact the main key pathogens in CF, that is, *P. aeruginosa, S. aureus*, and *A. fumigatus* in this study. Interestingly, a significant reduction in the inflammatory parameters was identified the year after lumacaftor/ivacaftor initiation. In terms of clinical outcomes, our study also showed a beneficial effect of lumacaftor/ivacaftor with an improvement on lung function and nutritional status.

CFTR modulators are the only available therapies targeting the basic defect in CF. Improving the CFTR function could theoretically improve the CF airways’ ability to clear microorganisms leading to a decrease in bacterial and fungal loads, a hypothesis that several researchers have investigated. Our study showed some changes in the microbiological outcomes 1 year after initiation of lumacaftor/ivacaftor which is in line with former study performing next generation sequencing of the 16S rRNA gene to analyze the microbial composition.^11^ On the other hand, other studies demonstrated no changes in the bacterial composition or the pathogen abundance.^9,10^ Regarding the most relevant pathogen in CF, *P. aeruginosa* no significant changes in the abundance after lumacaftor/ivacaftor treatment was reported.^9,10^ With respect to the difference in the microbiological detection methods, this is in line with our result. Compared to other CFTR modulators, several studies investigated the impact of ivacaftor on the microbiological outcomes with inconsistent findings. In contrast to our findings, pwCF with G551D mutation treated with ivacaftor exhibited a significant reduction of *P aeruginosa* and *A. fumigatus*.^13,28,29^ Other studies have not been able to confirm these results.^23,24^ Recent studies on elexacaftor/tezacaftor/ivacaftor, the most common and most effective CFTR modulator, showed a significant reduction in CF key pathogens; the limitation of these studies is the low number of participants.^18,19,30^

A possible explanation to the absence of an effect of lumacaftor/ivacaftor on the key pathogens can be the short follow-up period, which can be supported by a recently published study showing no alteration of the bacterial load nor of the isolation of *P. aeruginosa* 6 months after ivacaftor initiation.^23^ Further, in a 5-year follow-up study on ivacaftor, the authors reported that a clearance of *P. aeruginosa* occurred by the end of the study period.^28^

Regarding other relatively common CF bacteria, our study is the first to show a decrease in *S. maltophilia* with CFTR modulator treatment. *S. maltophilia* is a multidrug-resistant gram-negative bacteria reported as an independent risk factor for hospitalization due to lung exacerbations.^38^ Further, we found a reduced detection of *S. pneumoniae* with lumacaftor/ivacaftor. Similarly, Reznikov et al. reported an inhibitory effect of ivacaftor *in vitro* on the growth of clinical and laboratory *S. pneumoniae* strains.^15^ In the sensitivity test including patients who had greater than equal to three bacterial cultures annually, the slope of the prevalence of *Achromobacter* species was reduced after start with lumacaftor/ivacaftor. *Achromobacter* species is also a multidrug-resistant gram-negative bacterium associated with lung function decline and inflammation similar to the inflammation induced by *P. aeruginosa*.^39,40^ However, these findings should be interpreted with great caution due to the low prevalence of *S. pneumoniae* and *Achromobacter* species in our study cohort.

With respect to fungal outcomes, some studies investigated the effect of ivacaftor on *A. fumigatus* showing a decrease in prevalence, which we could not confirm in our study.^13,29^ One single study showed that treatment with elexacaftor/tezacaftor/ivacaftor was associated with a substantial reduction in isolation of *A. fumigatus*.^30^ The limitation of this study was the lack of data about the number of obtained sputum culture before and after initiation of elexacaftor/tezacaftor/ivacaftor treatment which may impact the isolation rate.

With the exception for *A. fumigatus*, fungi have not been studied in the era of CFTR modulators. As far as we know, the current study is the first to study fungi other than *A. fumigatus* and demonstrated an increase in the findings of *C. albicans, Penicillium* spp, and *S. apiospermum* after 1 year on lumacaftor/ivacaftor. The number of patients with isolated *S. apiospermum* was very low before and after lumacaftor/ivacaftor, and the results should therefore be interpreted with caution. *C. albicans* and *Penicillium* spp have not received much attention from researchers in the field and are traditionally considered as innocent colonizers.^41,42^ Two longitudinal studies along with our previous study reported an association between chronic colonization with *C. albicans* and lung function decline.^43–45^ *Penicillium*, the second most prevalent mold, is an environmental fungus and generally not considered as pathogenic in CF^31,32^ The possible explanation to the increase in these fungi could be the increased number of fungal cultures which consequently increased the probability to detect fungi. Further, the use of antifungal agents decreased after starting with lumacaftor/ivacaftor, which may explain the increase in the detection of *C. albicans.* It could also be argued that lumacaftor/ivacaftor may decrease the growth of some bacteria and consequently lead to less competition with fungi in the culture agar. Overall, our study demonstrated changes in the fungal outcomes, which is in contrast with recently published study on the mycobiota showing no changes on the fungal diversity and abundance 6 months after treatment with lumacaftor/ivacaftor.^10^

This study confirmed previously published studies on the impact of lumacaftor/ivacaftor on lung function and BMI. Furthermore, a reduction in the inflammatory parameters was observed. However, the serological response to *P. aeruginosa, S aureus*, and *A. fumigatus* did not alter after initiation of lumacaftor/ivacaftor possibly due to the short follow-up period. Regarding the treatment burden, our study showed a reduction in the antifungal treatment after initiation of the treatment. As far as we know, our study is the first to determine the use of antifungal treatment in pwCF treated with CFTR modulators. The possible reason for this reduction is the drug–drug interaction between lumacaftor/ivacaftor and azoles and is not related to reduction in the findings of *A. fumigatus* (the most common indication for antifungal treatment). In line with a previous study, iv antibiotic courses and treatment with oral antibiotics continuously reduced.^7^ Inhaled antibiotic, hypertonic saline and rhDNAse did not change after lumacaftor/ivacaftor treatment in this study, which was in line with the clinical intention to keep the maintenance treatment the same during the first year of lumacaftor/ivacaftor treatment.

Our study has several strengths. First, the relatively high number of collected respiratory cultures per patient and year reduces the risk of misclassification of the outcome. Second, the number of the annual bacterial cultures before and after lumacaftor/ivacaftor initiation was consistent providing data for a solid comparison.

In almost all developed countries, lumacaftor/ivacaftor has been replaced with elexacaftor/tezacaftor/ivacaftor, the next-generation and highly effective CFTR modulator.^46^ Studies on elexacaftor/tezacaftor/ivacaftor reported an exceptional effect on essential clinical CF features in pwCF both homozygous and heterozygous for F508del.^47,48^ Novel studies demonstrated shifting toward healthier but not normal airway.^19,21,22^ PwCF treated with elexacaftor/tezacaftor/ivacaftor exhibited a reduction in the exacerbation rate with 63%.^49^ Given the reduction in antibacterial treatment, it is essential to endure the monitoring of the microbiological outcomes. However, treatment with highly effective CFTR modulators reduces sputum production and makes it more difficult to expectorate sputum samples to culture. Therefore, it is urgent to establish appropriate microbiological detection methods in this group of patients. Further, the possible anti-inflammatory and antimicrobial effects of highly effective CFTR modulators are of great interest. The pathogenicity of restoring CFTR and its implication in airways’ environment and in the innate and adaptive immune response are needed to be profoundly elucidated. Detailed knowledge on these aspects may pave the way for more personalized treatment strategies for pwCF.

## Limitations

This study was hampered by some limitations. The main weakness of the study is the short follow-up period. Longer follow-up period may reveal more changes on the microbiological outcomes.^28^ A further limitation is that although all available data from the three CF centers in Sweden were used in the present study, the population is quite small, which negatively impacts the statistical power of analyses. Moreover, the number of patients with less common pathogens was low; therefore, study results should be interpreted with caution. Finally, micro- and mycobiological analyses were based on the conventional detection method, which is less sensitive than molecular analysis such as polymerase chain reaction and next-generation sequencing of the 16S rRNA gene.

## Conclusion

The current study demonstrated modest but significant improvement in lung function, BMI in adults, BMI *z*-score in children, reduction of the antibacterial and antifungal treatments 1 year after starting lumacaftor/ivacaftor, and less prevalence of *S. pneumoniae* and *S. maltophilia*. However, no significant changes in the outcomes of key CF pathogens such as *P. aeruginosa, S. aureus*, or *A. fumigatus* were observed.

## Supplemental Material

sj-docx-1-tar-10.1177_17534666241254090 – Supplemental material for Impact of lumacaftor/ivacaftor on the bacterial and fungal respiratory pathogens in cystic fibrosis: a prospective multicenter cohort study in Sweden Supplemental material, sj-docx-1-tar-10.1177_17534666241254090 for Impact of lumacaftor/ivacaftor on the bacterial and fungal respiratory pathogens in cystic fibrosis: a prospective multicenter cohort study in Sweden by Mahasin Al Shakirchi, Kimmo Sorjonen, Lena Hjelte, Lena Klingspor, Peter Bergman, Petrea Ericson, Marcus Svedberg, Ulrika Lindberg, Christine Hansen and Isabelle de Monestrol in Therapeutic Advances in Respiratory Disease

## Declarations

**Ethics approval and consent to participate:** The study was conducted according to the guidelines of the Declaration of Helsinki. Two ethical approvals are linked to this study. The one for Stockholm CF participants with reference number 2017/1493-3 allowed reviewing medical records and obtaining blood samples beyond the local routines (*A. fumigatus* specific antibodies). All included patients from Stockholm CF center or their guardians signed an informed consent form. Lumacaftor/ivacaftor was approved initially from 12 years of age at the time of application in 2017. The age limit was lowered to 6 years in 2018. An amendment was approved with reference number 2018/2006-32 to include patients from 6 years of age. For participant from Gothenburg and Lund, the study was performed as registry-based study and had an approval from the Regional Ethical Review Board in Stockholm, reference number 2019-00417, an ethical approval to use anonymous data from the registry for research purposes without the need of further consent.

**Consent for publication:** Not applicable.

**Availability of data and materials:** Not applicable.
